# FIB-4: A screening tool for advanced liver fibrosis in a cohort of subjects participating in a primary care weight-loss program

**DOI:** 10.1371/journal.pone.0333490

**Published:** 2025-10-07

**Authors:** Victoria Mignot, Odile Fabre, Rémy Legrand, Sébastien Bailly, Charlotte Costentin

**Affiliations:** 1 Department of Hepato-Gastroenterology and Digestive Oncology, Univ. Grenoble Alpes, CHU Grenoble Alpes, Grenoble, France; 2 Groupe Ethique & Santé – Head office, Aubagne, France; 3 University of Grenoble Alpes, Laboratory, INSERM U1300, Grenoble Alpes University Hospital, Grenoble, France; 4 Univ. Grenoble Alpes, Institute for Advanced Biosciences, CNRS UMR 5309-INSERM U1209, Grenoble, France; Cliniques Universitaires Saint-Luc, BELGIUM

## Abstract

Systematic screening for liver fibrosis using FIB-4 score is recommended in primary care for patients with chronic liver disease risk factors. This study assesses the prevalence and characteristics of patients at risk for advanced fibrosis in a weight loss program. This multicenter retrospective cohort study includes obese and overweight subjects participating in a weight loss program across 110 French centers. 34 510 participants with baseline FIB-4 available were included, predominantly women (78.3%), median age of 54 years, 70% obese. Baseline FIB-4 values were <1.3, 1.3–2.67 or >2.67 in 80.9%, 18.1% and 1% of the participants, respectively. When moving from the lower risk category (<1.3) to the highest (>2.67), the rates of metabolic comorbidities such as diabetes rose (from 3.2% to 13.3%). After 5 (3–7) months, all anthropometric parameters improved. A follow-up FIB-4 value was available in 20.7% participants. Among high-risk, 43% changed classes, 4.6% moving to the lower risk-category.

## Introduction

Obesity is a major public health issue. Worldwide, 60% of the population is overweight, 25% of whom are obese, with increased overall mortality and risk of co-morbidities, including liver diseases [1,2]. Indeed, obesity is an independent risk factor for metabolic-associated steatotic liver disease (MASLD). Individuals living with obesity are 3.5 times more likely to develop MASLD, and there is a dose-dependent relationship with Body Mass Index (BMI) [3,4]. MASLD has become one of the leading causes of chronic liver disease in industrialized countries. While around 25% of the world’s population currently lives with MASLD [5], the prevalence of MASLD in individuals living with obesity is much higher, around 75% according to the Dionysos study [6]. This proportion rises to 90% in patients with BMI greater than 40 kg/m^2^ [7]. Conversely, when considering the rate of obesity in MASLD, a meta-analysis estimated the overall prevalence of 51.3%. Thus, individuals living with obesity account for a large part of MASLD patients [8]. An increase in the prevalence of MASLD is expected in the coming years due to the global metabolic epidemic [9,10] and MASLD is one of the leading causes of cirrhosis and hepatocellular carcinoma (HCC) [11]. The risk of cirrhosis is increased by 3.2-fold in the presence of MASLD and the risk of HCC by 6.5 [12]. Liver fibrosis is the only determinant of liver-related mortality, particularly at the advanced stage (F3-F4) [13,14]. Unfortunately, in 75% of cases, cirrhosis is diagnosed at a decompensated stage, and HCC at an advanced stage not eligible for curative treatment, negatively impacting prognosis [15,16,17,18]. Late diagnosis prevents the implementation of measures to limit the progression of fibrosis and HCC screening programs [19]. Therefore, early diagnosis of liver fibrosis is critical to improve the prognosis of chronic liver disease in general and HCC in particular [20].

International societies have issued recommendations for systematic screening for liver fibrosis in primary care whenever a risk factor for chronic liver disease is identified. FIB-4 has been selected as the first-line tool to identify patients at risk of advanced fibrosis requiring additional liver fibrosis assessment, with a threshold at FIB-4 > 1.3 [21]. FIB-4 is a simple test, based on levels of aspartate aminotransferase (AST), alanine transaminase (ALT), platelets and age with no extra cost [22].

The RNPC® (Rééducation Nutritionnelle Psycho-Comportementale, *i.e.,* Nutritional Psycho-Behavioural Reeducation) French program is a weight-loss program implemented in 110 centers in France, whose effectiveness in weight loss has been previously reported [23]. Weight loss is a fundamental strategy for preventing MASLD in obese patients [24,25] and previous studies have reported histological improvement in the liver in obese MASLD patients with weight loss [26].

The aim of this study was to assess the prevalence and characteristics of patients at risk of advanced fibrosis in a cohort of overweight and obese subjects participating in the RNPC® program, and to assess variations in FIB-4 during weight loss.

## Materials and methods

### Study design

This is a retrospective multicenter cohort study involving overweight and obese subjects who participated in a weight-loss program carried out in each of the 110 RNPC® centers throughout France, with the agreement of their physician [23]. The RNPC® program is a three-stage weight-loss program including: (1) a rapid weight-loss phase (two to six months), (2) a stabilization phase during which energy intake is gradually increased, duration depending on the weight-loss phase (1 week per kg lost in the initial phase), and (3) a maintenance phase during which energy balance is achieved. Participants were included from January 1^st^ 2017 to June 09^th^ 2024.

The study protocol complied with the 1975 Declaration of Helsinki. All data used for this study were anonymous data from a database declared to the French authority Commission Nationale de l’Informatique et des Libertés (CNIL) under the number 1406514. Informed consent was obtained from all subjects and/or their legal guardian(s). Due to the retrospective nature of the study, the scientific committee of RNPC waived the need of obtaining approval.

### Population

RNPC® participants aged 18 and over were included in the study as soon as an entry biology work-up allowing FIB-4 calculation was available.

### Data collection

Data were collected systematically and consistently at each RNPC® center, using the same in-house electronic medical record (RNPC PILOT). Data collected included patient characteristics (age, gender), anthropometric data (weight, body mass index, waist circumference (WC), percentage of body fat, muscle mass and body water), habitus (smoking habits), main comorbidities, drug treatments, biological data (liver biology, complete blood count, complete ionogram, creatinine, urea, thyroid-stimulating hormone, lipid biology, glucose, Hb1Ac, insulin) and calculation of liver parameters: FIB-4 [22] and Fatty Liver Index (FLI) [27], in patients with required variables (age, AST, ALT and platelets for FIB-4, and BMI, Gamma-glutamyl transferase, WC and triglycerides for FLI).

The FIB-4 result was divided into categories: low risk of significant hepatic fibrosis if FIB-4 < 1.3; moderate risk if FIB-4 [1.3–2.67] and high risk if FIB-4 > 2.67.

Presence of steatosis was retained if FLI was greater to 60.

Data were collected at initial assessment and at the end of the program. Body weight, body water, muscle mass and fat mass percentages were measured using a calibrated bioelectrical impedance scale (BG 51 XXL, Beurer, Ulm, Germany).

Metabolic syndrome has been defined according to the International Diabetes Federation (IDF) by the presence of a WC ≥ 94 cm in men, ≥ 80 cm in women, or a BMI ≥ 30, associated with at least 2 of the following other criteria: fasting blood glucose ≥1 g/L or type 2 diabetes or antidiabetic treatment, triglycerides ≥1.5 g/L or specific treatment for this lipid disorder, high density lipoproteins <0.4 g/L in men or <0.5 g/L in women or specific treatment for this lipid disorder, blood pressure ≥130/85 mmHg or antihypertensive treatment [28]. Metabolic syndrome was considered when the above-mentioned criteria were found in the participant’s medical record. The diagnoses of diabetes, sleep apnea syndrome (SAS) and clinical diagnosis of MASLD were considered present if they were notified in the participant’s medical record.

Tobacco consumption was considered present if active at the time of program entry.

Alcohol consumption prior to program entry was not recorded, and participants were asked not to drink alcohol during the whole duration of the program.

### Endpoints

The primary endpoint was the number of patients with FIB-4 > 2.67 in the study population.

### Statistical analysis

Variables were described as median and interquartile range for quantitative variables, and as number and percentage for qualitative variables. Comparisons between groups were performed using Chi square test for qualitative variables and Kruskall-Wallis test for quantitative variables. A Bonferroni correction was performed for multiple comparisons.

To assess the evolution of patient characteristics over time, a linear regression was performed for quantitative values with an adjustment on age, sex, initial weight and duration between measures. For qualitative values, a logistic regression model was used for binary variable (metabolic syndrome and metabolic steatohepatitis risk) and an ordinal regression model was used for FIB-4 categories with the same adjustment variables. Models were performed on complete case analysis.

Statistical analyses were performed using SAS v9.4. A p-value threshold of 0.05 was considered significant.

### Missing data

Due to the low number of missing values, analyses were performed on complete cases.

## Results

### Flow chart

Of the 88,922 adult participants in the RNPC® program during the study period, of whom 34,510 (38.8%) had the variables required to calculate an initial FIB-4 score and were included in the analysis. Participants had a median follow-up of 5 months [IQR: 3–7] in the program. Of the 34,510 included participants with an initial FIB-4, 8,788 (25.5%) had at least one FIB-4 calculated during follow-up.

### Study population

Regarding the 34,510 patients with an initial FIB-4 score, there were mainly women (78.2%), with a median age of 54 years [44−63], a median initial BMI of 32.6 [29.2–36.4] kg/m^2^ (70% with BMI ≥ 30) and median initial WC of 107 cm [97−117] (**Table 1**). Initial FIB-4 was < 1.3 in 27,920 participants (80.9%) and >2.67 in 346 (1%). Participants had diabetes in 4% of the cases, metabolic syndrome in 40.6% and SAS in 17.3%. In all, 12,401 participants/ 34,510 had 1 risk factor for MASLD (35.9%) and 13,859 had 2 risk factors (40.1%). The Fatty Liver Index (FLI) was available for 17,626 participants, of whom steatosis defined by FLI > 60 was present in 12,352 subjects (70,1%). A diagnosis of MASLD was identified at program entry for 2,156 (6.2%) participants.

**Table 1:** Patient initial characteristics (whole population and according to FIB-4 score categories at baseline) (N = 34,510).

Variable	All population N = 34,510	FIB-4 < 1.3 N = 27,920 (80.9%)	FIB-4 [1.3–2.67] N = 6,252 (18.1%)	FIB-4 ≥ 2.67 N = 338 (1%)	Missing
Age (years)	54 [44-63]	51 [42-59]	66 [59-72]	70 [63-75]^*#^	0
Female (%)	26998 (78.2)	22558 (80.8)	4263 (68.2)^*^	177 (52.4)^* #^	0
Weight (kg)	89.3 [78.2-102.4]	89.2 [78.2-102]	89.8 [77.8-103.8]	96.9 [83.8-109]^* #^	10
BMI (kg/m^2^)	32.6 [29.2-36.4]	32.5 [29.2-36.4]	32.7 [29.2-36.6]	34.3 [30.5-37.5]^* #^	275
WC (cm)	107 [97-117]	106 [97-116]	110 [100-120]^*^	117 [107-125]^* #^	163
Fat mass (%)	40.8 [36.8-44.7]	40.7 [36.7-44.5]	41.2 [37.1-45.3]^*^	41.4 [37-45.2]	2,110
Muscle mass (%)	29.1 [27.1-31.9]	29.4 [27.4-32.1]	28 [25.6-31.3]^*^	28.1 [25.8-32.6]^*^	2,703
Body water (%)	43 [40-45.9]	43.1 [40.1-45.9]	42.5 [39.5-45.5]^*^	42.4 [39.6-45.6]	2,187
Smoking (%)	4,201 (12.2)	3763 (13.5)	412 (6.6)^*^	26 (7.7)^*^	0
SAS (%)	6,099 (17.7)	4276 (15.3)	1700 (27.2)^*^	123 (36.4)^* #^	0
Diabetes (%)	1,386 (4)	899 (3.2)	442 (7.1)^*^	45 (13.3)^* #^	0
Metabolic syndrome (%)	13,744 (40.6)	10618 (38.8)	2927 (47.7)^*^	199 (61)^* #^	660
AST (UI/L)	22 [18-27]	21 [17-26]	26 [22-33]	45 [29-72]^* #^	8
ALT (UI/L)	24 [18-36]	24 [17-35]	25 [18-37]^*^	35 [21-61.5]^* #^	8
Platelets (G/L)	265 [229-306]	277 [243-315]	216 [189-244]	166 [137-203]^* #^	141
FLI > 60 (%)	12,352 (70.1)	9814 (69)	2414 (74.3)^*^	124 (86.7)^* #^	16,892
MASLD diagnosis (%)	2,154 (6.2)	1507 (5.4)	570 (9.1)^*^	77 (22.8)^* #^	0

Median [IQR] or N (%)

*significantly different from FIB-4 < 1.3 group after correction for multiple tests

#significantly different from FIB-4 [1.3–2.67[group after correction for multiple tests

### Initial characteristics of participants by FIB-4 category

When moving from the low-risk category (FIB-4 < 1.3) to the high-risk category (FIB-4 > 2.67), prevalence of female participants significantly decreased from 80.8% to 52.4%, weight increased from 89.2 kg [78.2−102] to 96.9 kg [83.8−109], and WC from 106 cm [97−116] to 117 cm [107−125] (**Table 1**). Metabolic comorbidities were also more frequent: metabolic syndrome (38.8% to 61%), diabetes (3.2% to 13.3%) and SAS (15.3% to 36.4%) (p < 0.01). FLI 60 rates also significantly increased, from 69% to 86.7%. Similar trends were observed in the sub-population of patients with a follow-up FIB-4 available (**Table 2**).

**Table 2:** Patient initial characteristics in the population of patients with 2 FIB-4 scores available (whole population and according to FIB-4 score categories at baseline) (N = 8,788).

Variable	All population N = 8,788	FIB-4 < 1.3 N = 6902 (78.5%)	FIB-4 [1.3–2.67] N = 1796 (20.4%)	FIB-4 ≥ 2.67 N = 90 (1%)	Missing
Age (years)	56 [48-64]	53 [45-61]	66 [60-72]	69 [64-76]^*#^	0
Female (%)	6751 (76.8)	5502 (79.7)	1203 (67)^*^	46 (51.1)^* #^	0
Weight (kg)	89.6 [78.8-102.4]	89.4 [78.8-101.8]	90.3 [78.6-104.1]	100.9 [86.2-112]^* #^	1
BMI (kg/m^2^)	32.7 [29.6-36.5]	32.7 [29.6-36.3]	32.9 [29.6-36.8]	35 [31.3-38]^* #^	16
WC (cm)	108 [99-117]	107 [98-116]	110 [101-121]^*^	119 [109-125]^* #^	37
Fat mass (%)	41.2 [37.4-45.1]	41.1 [37.4-44.9]	41.7 [37.7-45.6]^*^	42.3 [37.7-45.4]	516
Muscle mass (%)	28.7 [26.6-31.5]	28.9 [26.9-31.5]	28 [25.3-31.2]^*^	27.8 [25.7-32.9]	690
Body water (%)	42.7 [39.7-45.4]	42.8 [39.9-45.5]	42.2 [39.4-45.1]^*^	41.2 [39.4-44.9]	545
Smoking (%)	876 (10)	761 (11)	110 (6.1)^*^	5 (5.6)	0
SAS (%)	1726 (19.6)	4276 (15.3)	1700 (27.2)^*^	123 (36.4)^* #^	0
Diabetes (%)	307 (3.5)	196 (2.8)	102 (5.7)^*^	9 (10)^*^	0
Metabolic syndrome (%)	3884 (44.8)	10618 (38.8)	2927 (47.7)^*^	199 (61)^* #^	660
AST (UI/L)	22 [18-28]	21 [18-26]	26 [22-33]^*^	46.5 [29-72]^* #^	0
ALT (UI/L)	25 [18-37]	25 [18-36]	25 [18.5-38]	35.5 [19-66]^* #^	2
Platelets (G/L)	263 [226-303]	277 [242-313]	213 [187-241]	164.5 [139-196]^* #^	30
FLI > 60 (%)	3235 (73.9)	2492 (73.3)	702 (75.8)	41 (87.2)	4413
MASLD diagnosis (%)	670 (7.6)	447 (6.5)	205 (11.4)^*^	18 (20)^* #^	0

Median [IQR] or N (%)

*significantly different from FIB-4 < 1.3 group after correction for multiple tests

#significantly different from FIB-4 [1.3–2.67[group after correction for multiple test

### Evolution of FIB-4 and risks over time

After 5 (3–7) months of program follow-up, using a linear regression model adjusted on age, sex, initial weight and duration between measures we showed that there was a significant decrease in weight, BMI, WC over time. Regarding hepatic parameter, there was a significant decrease in ALT levels over time (**Table 3**). A follow-up FIB-4 value was available for 8,789 participants. The main change in FIB-4 proportion was observed in patients who move from <1.3 to [1.3–2.67] categories, but changes were not statistically significant overall (**Table 4**). There was a significant decrease over time in proportion of patients with a metabolic syndrome and in the proportion of participants at risk of metabolic-associated steatohepatitis defined by the presence of metabolic syndrome and elevated ALT ≥ 30 UI/L in men or 20 UI/L in women.

**Table 3:** Changes in participant continuous characteristics over the program.

Variable	Entry	Follow-up	Beta (SD)*	P-value
Weight (kg)	89.6 [78.8-102.4]	77 [68.1-88.9]	−0.0748 (0.013)	<.0001
BMI (kg/m^2^)	32.7 [29.6-36.5]	28.2 [25.5-31.5]	−0.0272 (0.0047)	<.0001
WC (cm)	108 [99-117]	94 [86-104]	−0.1237 (0.0132)	<.0001
Fat mass (%)	41.2 [37.4-45.1]	37 [33-41.2]	−0.0064 (0.0078)	0.4080
Muscle mass (%)	28.7 [26.6-31.5]	30 [27.7-33]	−0.0070 (0.0048)	0.1403
AST (UI/L)	22 [18-28]	20 [17-24]	−0.0195 (0.0166)	0.2390
ALT (UI/L)	25 [18-37]	21 [16-28]	−0.1015 (0.0277)	0.0002
Platelets (G/L)	263 [226-303]	248 [213-287]	0.0543 (0.0628)	0.3867
FLI > 60 (%)	82.4 [58.7-93.9]	42.2 [20.7-70.1]	−0.0983 (0.0612)	0.1084

*Results of linear regression model for follow-up value adjusted on age, sex, initial weight, initial value and duration between measures.

**Table 4:** Changes in participants categorical characteristics over the program.

Variable	Entry	Follow-up	OR [95% CI]*	P-value
Metabolic syndrome (%)	3884 (44.8)	1239 (15)	0.92 [0.89-0.93]	<.0001
Risk of MASLD (%)	2819 (32.3)	721 (8.5)	0.90 [0.87-0.92]	<.0001
FIB-4 (%)				
<1.3	6902 (78.5)	6600 (75.1)	1.01 [0.99-1.02]	0.2098
[1.3-2.67]	1796 (20.4)	2077 (23.6)		
>2.67	90 (1)	111 (1.3)		

*Result of logistic regression for metabolic syndrome and risk of metabolic steatohepatitis and ordinal logistic regression for FIB-4 categories to assess the effect of time on the evolution of both variables. Value adjusted on age, sex, initial weight, initial value and duration between measures.

## Discussion

Our study reports the prevalence and characteristics of patients at risk of advanced fibrosis in a cohort of overweight and obese subjects participating in the RNPC® program and investigates the changes in the FIB-4 index over the time. Among the 34,518 participants who had a FIB-4 available at entry, the majority (78,1%) had at least one risk factor for advanced fibrosis. Our cohort of participants differs from the obese population in France, notably in its high proportion of women. Recent data from the Constances cohort (28,895 participants aged 30–69) indicate a prevalence of obesity of 15.8% in men and 15.6% in women [29]. In addition, the proportion of participants with diabetes in our study is lower than prevalence of diabetes in France [30].

Regarding liver health in our study, the FLI was available for 12,454 participants, of whom steatosis defined by FLI > 60 was present in 66%. Initial FIB-4 was > 2.67 in 346 participants (1%). These data are consistent with figures drawn from the Constances cohort, in which the prevalence of MASLD defined by an FLI > 60 was 79.1% in obese subjects and 62.4% in diabetic subjects [31]. In this same cohort, the rate of advanced fibrosis was 2.5% in obese subjects (defined by a Forns Index (FI) >6.9), which is slightly higher than in our study (1% with a FIB-4 > 2.67). The relatively low prevalence of high-risk FIB-4 scores in our cohort may be partly explained by this unexpectedly low prevalence of type 2 diabetes mellitus, a major risk factor for advanced liver fibrosis.

In our study, the diagnosis of MASLD was identified at program entry for 6.2% participants, which is much lower than the 66% MASLD suggested by an FLI > 60. This underlines low awareness of MASLD among practitioners. Indeed, in a survey sent to 133 UK diabetologists, only 5.7% used a non-invasive algorithm to assess the severity of a fatty liver disease [32]. Another survey of 178 French diabetologists showed that 59% underestimated the prevalence of chronic liver disease [33]. Moreover, patient awareness about MASLD is low. In an American study of over 10,000 adults, 96% of adults with MASLD were unaware that they had liver disease [34].

When moving from the low-risk category (FIB-4 < 1.3) to the high-risk category (FIB-4 > 2.67), prevalence of male increased from 19.2% to 48%, weight increased from 89.2 kg to 96.9 kg, diabetes from 3.2% to 13.3%, and metabolic syndrome from 38.8% to 61%. These results are consistent with the well-known risk factors for hepatic fibrosis [31, 8]. Interestingly SAS increased from 15.3% to 36.4% moving from the low-risk category (FIB-4 < 1.3) to the high-risk category (FIB-4 > 2.67). Although not clearly identified as a risk factor for liver fibrosis in the literature, several studies have shown that it is independently associated with increased liver stiffness in patients with metabolic comorbidities [35,36].

An additional consideration when interpreting the FIB-4 score is the influence of age, which is directly included in the formula. As a result, FIB-4 values naturally increase with age, potentially leading to false positives for advanced fibrosis in older adults. In our cohort, participants with a FIB-4 > 2.67 were significantly older than those with a FIB-4 < 1.3, supporting this concern. Indeed, the EASL 2021 guidelines acknowledge that specificity of FIB-4 is reduced in those over 65 years, and although higher thresholds have been proposed for this age group, they remain unvalidated and are not recommended in current EASL guidance [21].

In our study, after 5 (3–7) months of program follow-up, there was a significant decrease in weight, BMI, WC, metabolic syndrome and ALT levels over time. In contrast, for participants who had a follow-up FIB-4 value available, there was no significant change in FIB-4 category. The absence of a decrease in FIB-4 while metabolic parameters improve over time should not be interpreted as a lack of efficacy on liver health. Indeed, weight loss and improved metabolic parameters in metabolic dysfunction-associated steatohepatitis (MASH) have been associated with improved liver histology in various studies [37,38]. Moreover, weight loss is recommended as the first-line treatment for this pathology [21]. Variations in FIB-4 over time were studied by Hagstrom, who found that repeated FIB-4 testing within 5 years improved the identification of individuals at increased risk of severe liver disease in the general population. [39]. However, variations in FIB-4 during weight loss have received little attention. Kawai et al also investigated whether the FIB-4 index improved during weight reduction therapy [40]. A negative correlation was observed between weight loss and FIB-4 after 3 months, but not after 6 months, whereas a positive correlation was noted after 12 months, demonstrating that while FIB-4 is useful for assessing liver fibrosis initially, it loses its usefulness during weight loss.

One important limitation of our study is the relatively low proportion of participants with sufficient data to calculate baseline FIB-4 (38.8%), and an even smaller subset with follow-up measurements (9.9%). This reflects the inherent challenges of retrospective data collection in routine clinical practice. Moreover, alcohol consumption prior to program entry was not recorded, while it is a major risk factor for chronic liver disease, frequently associated with MAFLD and contributing in an intricate way to the pathogenesis of the liver disease [41]. The main limitation of our study is the lack of confirmation of advanced fibrosis with a second line fibrosis test. However, in a study from France, ELF confirmed advanced fibrosis in 80% of patients with FIB-4 > 2.67, suggesting FIB-4 > 2.67 alone should translate into referral to gastroenterology specialist for follow-up [42].

In conclusion, in a cohort of overweight and obese patients from the primary care setting, prevalence of patients at risk for advanced fibrosis according to initial FIB-4 > 1.3 was 19.1%. Higher-risk participants (FIB-4 > 2.67, 1%) displayed higher rates of metabolic comorbidities. General practitioners and nutrition professionals are important assets to implement the two-step algorithm to screen for advanced fibrosis in patients at risk. Efforts should be made to improve the care pathway to the second line non-invasive fibrosis tests.

## Supporting information

S1 Table Comparison of patients’ baseline characteristics with or without available FIB4 value during follow-up. (CSV)

S2 Table Anonymized dataset for the main results of the manuscript. The dataset include the following variables: ID: patient identifier; age: age in years;sexe: sex (male/female); poids_BS: baseline weight (kg); FIB4_1: initial FIB-4 measure; FIB4_2: final FIB-4 measure; duration_days: number of days between baseline and final measures; synd_met_1: metabolic syndrome at baseline (1 = present, 0 = absent); synd_met_2: metabolic syndrome at final assessment (1 = present, 0 = absent); shr_1: steatohepatic risk at baseline (binary variable); shr_2: steatohepatic risk at final assessment (binary variable).
(DOCX)

## Abbreviations

ALT: alanine transaminase
AST: aspartate aminotransferase
BMI: body mass index
CNIL: Commission Nationale de l’Informatique et des Libertés
FI: Forn Index
FLI: Fatty Liver Index
HCC: hepatocellular carcinoma
IDF: International Diabetes Federation;
MASH: metabolic dysfunction-associated steatohepatitis
MASLD: metabolic-associated steatotic liver disease
RNPC®: Rééducation Nutritionnelle Psycho-Comportementale
SAS: sleep apnea syndrome
W.C.: waist circumference
