# Supplementary material for: FIB-4: A screening tool for advanced liver fibrosis in a cohort of subjects participating in a primary care weight-loss program
Source: PLoS One. 2025 Oct 7;20(10):e0333490. doi: 10.1371/journal.pone.0333490 (PMC12503257; doi:10.1371/journal.pone.0333490)
Supplement: S2 Table — The dataset include the following variables: ID: patient identifier; age: age in years;sexe: sex (male/female); poids_BS: baseline weight (kg); FIB4_1: initial FIB-4 measure; FIB4_2: final FIB-4 measure; duration_days: number of days between baseline and final measures; synd_met_1: metabolic syndrome at baseline (1 = present, 0 = absent); synd_met_2: metabolic syndrome at final assessment (1 = present, 0 = absent); shr_1: steatohepatic risk at baseline (binary variable); shr_2: steatohepatic risk at final assessment (binary variable). (DOCX) [file pone.0333490.s002.docx]

| Variable | Without FIB4 measures  N=88,922 | With FIB4 measures  N=34,518 | Missing |
| --- | --- | --- | --- |
| Age (years) | 55 [45-64] | 54 [44-63] | 0 |
| Female (%) | 41500 (76.3) | 26998 (78.2) | 0 |
| Weight (kg) | 88.5 [77.2-101.8] | 89.3 [78.2-102.4] | 38 |
| BMI (kg/m^2^) | 32.2 [28.9-36.1] | 32.6 [29.2-36.4] | 16 |
| WC (cm) | 106 [97-116] | 107 [97-117] | 458 |
| Fat mass (%) | 40.4 [36.2-44.4] | 40.8 [36.8-44.7] | 5950 |
| Muscle mass (%) | 29.4 [27-32.5] | 29.1 [27.1-31.9] | 7300 |
| Body water (%) | 43.1 [40-46.2] | 43 [40-45.9] | 8032 |
| Smoking (%) | 6305 (12) | 4201 (12.2) | 0 |

**Supporting Table 1: Comparison of patients’ baseline characteristics with or without available FIB4 value during follow-up**

Median [IQR] or N (%)
